# Simultaneous Bilateral Quadriceps Tendon Rupture After Low-Energy Trauma in a 78-Year-Old Woman: Delayed Diagnosis, Ultrasound Confirmation, and Single-Stage Repair

**DOI:** 10.7759/cureus.109787

**Published:** 2026-05-28

**Authors:** Georgi Luchev, Maria Piagkou, Ahmed Al-Sadek, Svetoslav A Slavchev, Nikolay Yordanov, Dimcho Hadziev, Georgi P Georgiev

**Affiliations:** 1 Department of Orthopaedics and Traumatology, University Hospital Queen Giovanna - ISUL, Medical University of Sofia, Sofia, BGR; 2 Department of Anatomy, National and Kapodistrian University of Athens, Athens, GRC

**Keywords:** bilateral quadriceps tendon rupture, delayed diagnosis, simultaneous, surgical repair, ultrasound imaging

## Abstract

Simultaneous bilateral quadriceps tendon rupture (BQTR) is an uncommon extensor mechanism injury, with particularly few reports in elderly women without major systemic risk factors. We present the case of a 78-year-old woman who sustained sequential BQTRs after a low-energy fall. The initial assessment at another facility attributed her symptoms to knee osteoarthritis, resulting in a one-week delay in diagnosis. Re-evaluation revealed bilateral loss of active knee extension and palpable suprapatellar defects, and musculoskeletal ultrasound confirmed complete bilateral rupture. The patient underwent single-stage bilateral surgical repair using transosseous patellar tunnels and high-strength sutures, performed by two surgical teams simultaneously. Mobilization and supervised rehabilitation were started on the first postoperative day. At follow-up, the patient regained functional ambulation with satisfactory knee motion. This case highlights a clinically relevant diagnostic pitfall in elderly patients with pre-existing degenerative knee disease and demonstrates that simultaneous bilateral repair can be feasible even after a short diagnostic delay.

## Introduction

Simultaneous bilateral quadriceps tendon rupture (BQTR) represents a rare disruption of the knee extensor mechanism. The reported incidence of quadriceps tendon rupture (QTR) is approximately 1.3-1.5 per 100,000 persons annually, whereas bilateral cases constitute only a small subset of these injuries [1,2]. Most published cases involve middle-aged men and are associated with systemic conditions that predispose to tendon degeneration, including chronic kidney disease, diabetes mellitus, secondary hyperparathyroidism, inflammatory disorders, obesity, and long-term corticosteroid use [1,3,4]. Reports involving elderly women without clear systemic risk factors remain limited [5,6].

Diagnostic delay is a recurring problem in BQTR, particularly in older patients. The presentation may be confused with more common causes of impaired mobility, including pain from degenerative joint disease, neurological impairment, or generalized frailty [2,7]. This is especially relevant when pre-existing knee osteoarthritis is present, because loss of active extension may be incorrectly interpreted as pain-limited motion rather than extensor mechanism failure. A missed diagnosis can prolong immobilization and contribute to quadriceps atrophy, stiffness, and functional decline [1,4].

Complete QTR is generally treated surgically, with better outcomes reported when repair is performed in the acute setting [5,8]. Restoration of extensor mechanism continuity permits structured rehabilitation and may prevent the need for more complex reconstruction in chronic or neglected ruptures [2,4]. In geriatric patients, the treatment strategy must also minimize immobilization-related morbidity while protecting the repair during early healing [6].

The present report describes a 78-year-old woman with sequential BQTR after low-energy trauma, in whom the diagnosis was established clinically and confirmed by musculoskeletal ultrasound (US) after an initial missed diagnosis. The case is discussed with emphasis on diagnostic pitfalls, the practical role of US, and the rationale for single-stage bilateral repair in an elderly patient.

## Case presentation

A 78-year-old woman presented to the Emergency Department after a fall from standing height. She reported initially landing on one knee, followed by immediate pain in the suprapatellar region. While attempting to stand and transfer weight to the contralateral limb, she experienced sudden pain above the opposite patella, fell again, and was subsequently unable to stand or walk. Prior to the injury, the patient had been living independently and had been ambulatory without walking aids. She had no previous history of knee trauma or prior knee surgery. Apart from a BMI of 33 kg/m^2^, she did not have any chronic diseases that might increase the risk of spontaneous tendon ruptures.

The first orthopedic assessment did not identify the extensor mechanism injury. On repeat evaluation one week later, a persistent inability to actively extend either knee prompted a targeted examination, which revealed bilateral suprapatellar depressions consistent with quadriceps tendon defects and established the diagnosis of simultaneous BQTR.

Physical examination demonstrated swelling and tenderness in the suprapatellar region of both knees. Active knee extension was absent bilaterally, and straight-leg raise testing could not be performed on either side. Palpation identified a distinct defect proximal to the superior pole of each patella, findings consistent with BQTR.

**Figure 1:**
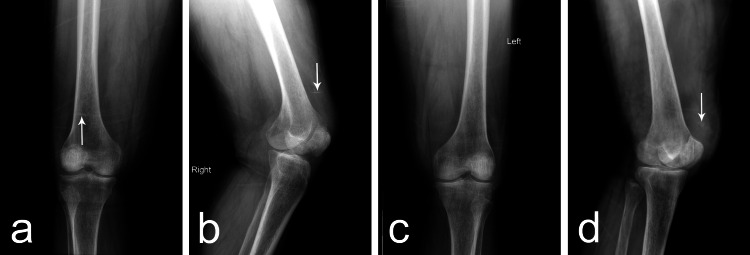
Preoperative radiographs of both knees. (a) Anteroposterior and (b) lateral views of the right knee. (c) Anteroposterior and (d) lateral views of the left knee. Moderate bilateral degenerative changes consistent with Kellgren-Lawrence grade 2 osteoarthritis are present. Small avulsion fragments adjacent to the superior poles of the patellae (arrows), together with bilateral patella baja, are visible and represent subtle but important radiographic signs of quadriceps tendon rupture.

Preoperative plain radiographs showed moderate degenerative changes corresponding to Kellgren-Lawrence grade 2 osteoarthritis [9]. Indirect radiographic signs of extensor mechanism disruption were also present, including bilateral patella baja. In addition, a small avulsed osseous fragment adjacent to the superior pole of the patella was identified, further supporting the diagnosis (Figures 1a-1d).

**Figure 2:**
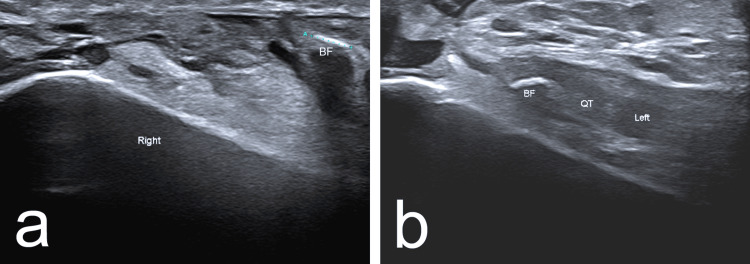
Longitudinal musculoskeletal ultrasound images of the suprapatellar region. (a) Right knee and (b) left knee demonstrating complete disruption of the quadriceps tendons with loss of normal fibrillar continuity and associated hematoma within the defect. Small, avulsed bone fragments are also visible adjacent to the superior patellar poles. BF: bone fragment; QT: quadriceps tendon

Musculoskeletal US of both knees was used as the confirmatory imaging modality. It demonstrated bilateral disruption of the normal fibrillar architecture of the QTs, with well-defined hypoechoic defects proximal to the superior poles of the patellae. The findings confirmed complete BQTR, and associated avulsion fragments were visible on both sides (Figure 2).

Additional findings included surrounding soft tissue edema and proximal tendon retraction. Dynamic assessment during attempted quadriceps contraction further demonstrated bilateral functional discontinuity of the extensor mechanism, supporting the diagnosis. Both patellar tendons remained intact.

Because both extensor mechanisms were disrupted and the patient was unable to ambulate, operative repair was indicated. Surgery was performed with the patient in the supine position under general anesthesia. Two independent orthopedic teams operated simultaneously, each treating one knee, to shorten operative time and reduce anesthesia exposure in an elderly patient with limited physiological reserve.

A standard anterior midline approach was used for the left knee. On the right side, a lateral parapatellar skin incision was selected because of prepatellar skin abrasions. Intraoperative findings confirmed complete BQTR at the superior poles of the patellae. After evacuation of hematoma and debridement of devitalized tissue, both tendons were prepared for reinsertion using No. 2 FiberWire sutures (Arthrex, Naples, Florida, USA). Three longitudinal transosseous tunnels were created in each patella.

**Figure 3:**
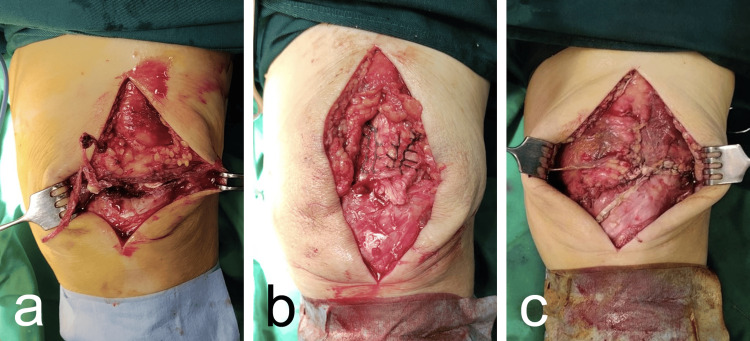
Intraoperative photographs of the left knee. (a) Complete quadriceps tendon rupture with retracted tendon edges exposed after surgical approach. (b) Tendon repair following placement of locking Krackow sutures and passage through transosseous patellar tunnels. (c) Final construct demonstrating secure reattachment of the quadriceps tendon to the superior pole of the patella with restoration of extensor mechanism continuity.

**Figure 4:**
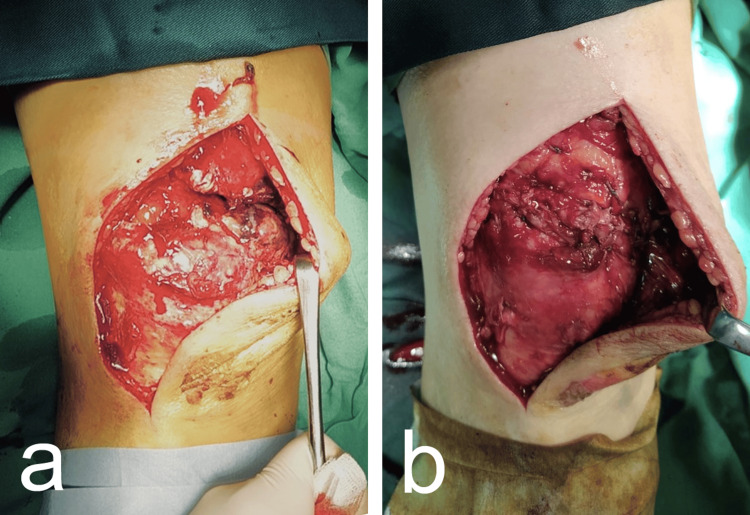
Intraoperative photographs of the right knee. (a) Surgical exposure demonstrating complete avulsion of the quadriceps tendon from the superior pole of the patella with disrupted extensor mechanism fibers. (b) Debridement and preparation of the tendon insertion site prior to transosseous repair using high-strength sutures.

The two surgical teams used different repair configurations. In the left knee (Figure 3), a double Krackow locking stitch was placed in the tendon, and the suture limbs were passed distally through the patellar tunnels and tied over the inferior pole. Retinacular tears were then repaired. In the right knee (Figure 4), the sutures were first passed through the patella from distal to proximal and then secured to the tendon using a full-thickness horizontal mattress configuration combined with multiple far-near/near-far fixation stitches. Additional running sutures were used to repair the retinacular defects as permitted by tissue quality.

All sutures were tied with the knees in full extension to restore tendon length and maintain appropriate tension. Intraoperative flexion testing using a drop-and-dangle maneuver demonstrated stable repairs without gapping or suture cut-through. Both wounds were irrigated and closed in layers without the placement of surgical drains.

Postoperatively, both repairs were protected using hinged knee braces, allowing 30° of flexion for the first 10 days, followed by weekly increases of 15° over the subsequent four weeks. Rehabilitation began on the first postoperative day and included supervised ambulation with protected full weight-bearing. Early physiotherapy emphasized safe bed-to-chair transfers, use of walking aids, prevention of complications related to immobilization, and maintenance of lower-limb activity through ankle pumps, gentle patellar mobilization, isometric quadriceps exercises, active hip motion, and controlled knee motion within the permitted range.

After postoperative day 30, the patient was gradually weaned from walking aids. The rehabilitation program was then advanced to include progressive resistance exercises, single-leg balance training, and gradual increases in knee range of motion during closed-chain activities. Standard venous thromboembolism prophylaxis was administered throughout hospitalization, and analgesics were provided as required.

**Figure 5:**
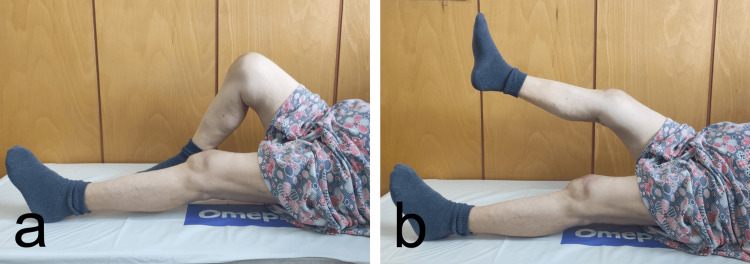
Postoperative clinical assessment of the right knee on postoperative day 55. (a) Knee flexion exceeding 90°. (b) Active straight-leg raise demonstrating near-complete active extension with minimal extensor lag and satisfactory quadriceps function.

**Figure 6:**
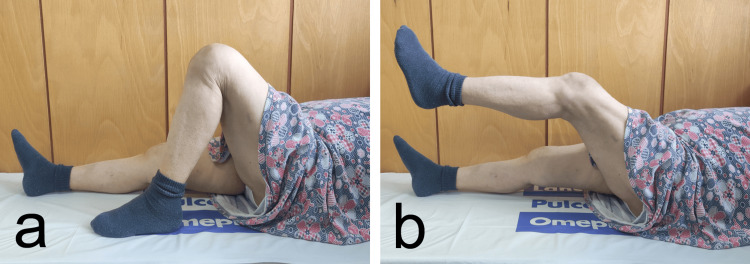
Clinical assessment of the left knee on postoperative day 55. (a) Knee flexion to approximately 120°. (b) Active straight-leg raise demonstrating residual extensor lag of approximately 25°, with preserved functional quadriceps activation.

At postoperative day 55, right knee flexion exceeded 90° (Figure 5a), while left knee flexion reached 120° (Figure 6a). The right knee demonstrated near-full active extension with minimal lag (Figure 5b). The left knee showed an extension lag of approximately 25° (Figure 6b). Lysholm Knee Scoring scale was applied to the patient, which showed 85 for the right knee and 79 for the left knee.

Because the patient lived alone, she did not have sufficient family support to participate in a structured outpatient rehabilitation program and, therefore, continued inpatient rehabilitation. Functionally, she was able to stand and ambulate with a walker, with ongoing gradual improvement. Overall, pain was minimal, with only mild discomfort in the left quadriceps during active extension and a subjective sensation of incomplete knee straightening.

No early postoperative complications, including wound dehiscence, infection, rerupture, or fixation failure, were observed. Overall, the early outcome was favorable, with restoration of functional mobility and satisfactory knee flexion, although persistent left-sided extensor lag remained present.

## Discussion

Epidemiology and clinical relevance

Simultaneous BQTR is most commonly reported in patients with systemic or metabolic conditions that predispose to tendon degeneration, including diabetes mellitus, chronic kidney disease, inflammatory disorders, obesity, and prolonged corticosteroid exposure [1,4,8]. Published series also demonstrate a marked male predominance, with most patients being middle-aged men. The elderly patient reports summarized in Table 1 follow this pattern, with few comparable cases in women and no major complications requiring revision surgery reported in the available cases.

**Table 1:** Published reports of simultaneous bilateral quadriceps tendon rupture (BQTR) in elderly patients. M: male; MRI: magnetic resonance imaging; EMG: electromyography; PET: polyethylene terephthalate; ROM: range of motion

Author	Age/Sex	Comorbidities/Risk Factors	Diagnostic Methods	Surgical Treatment	Postoperative Protocol	Follow-Up	Outcome
Clinical memoranda [10]	66/M	None reported	Physical examination	Transosseous bone tunnels	Not reported	Three months	90° knee flexion
Moriya et al. [11]	72/M; 69/M	None reported	Physical examination, X-ray, MRI	Transosseous sutures	Cast immobilization for six weeks	26 and 24 months	Full extension; flexion 120°-140°
Alkhatatba et al. [12]	70/M	None reported	Physical examination, X-ray, MRI	Two suture anchors per knee	Hinged braces locked in extension for one month	Four months	Mild residual functional limitations
Mihai et al. [13]	68/M	Diabetes mellitus, gout, hypertriglyceridemia, obesity	Physical examination, X-ray, MRI	Suture anchors with Krackow repair	Braces locked in extension for four weeks	Six months	Significant functional improvement
Utoyo et al. [14]	78/M	Diabetes mellitus	Physical examination, X-ray, MRI	Bunnell technique with two anchors and locked Krackow sutures	Brace locked in extension for one week	Six months	ROM 0°-125° bilaterally
Hewins et al. [15]	67/M	Neuromuscular weakness, spinal cord degeneration	Physical examination, X-ray, ultrasound	Allograft augmentation with bone tunnels and Krackow sutures	Off-loader braces	Two years	Independent ambulation
AlShaafi et al. [16]	67/M	Bilateral knee osteoarthritis, vitamin D deficiency, and diabetes mellitus	Physical examination, X-ray, ultrasound	Kessler repair with FiberTape	Knee immobilizers for six weeks	Three months	Independent walking; active straight-leg raise
Bove et al. [17]	65/M	Hypercholesterolemia	Physical examination, X-ray, MRI	Single-stage reconstruction with PET tape augmentation	Brace in extension for two weeks	12 months	Full extension; painless 120° flexion
Alpantaki et al. [18]	85/M	None reported	Physical examination, X-ray, MRI, EMG	Non-absorbable sutures through patellar tunnels	Cylinder cast for six weeks	Four months	ROM 0°-120° bilaterally
Ukpong-Dan and Dosani [19]	92/M	Hypertension, heart murmur	Physical examination, X-ray	Surgical repair (not specified)	Cylinder plaster casts	Not reported	Not reported
Lloyd [20]	77/M	Hematomyelia	Physical examination	Transosseous sutures	Not reported	One year	Walking regained
DePalma et al. [21]	66/M	Not reported	Not reported	Allograft reconstruction	Not reported	Two years	Excellent result
Umbel et al. [22]	66/M	Hypothyroidism	Not reported	Suture anchors	Not reported	One year	Full motion; 5° extension loss
Ellanti et al. [23]	67/M	None reported	Not reported	Not reported	Not reported	Not reported	Good postoperative function

Age-related degenerative tendon changes may have contributed to the injury mechanism in the present patient, but advanced age alone does not fully explain simultaneous bilateral failure of the quadriceps mechanism. This makes such cases useful for refining diagnostic awareness in older patients who may not have the classic systemic risk profile.

Diagnostic challenges and clinical examination

The classic presentation of QTR includes acute suprapatellar pain, inability to actively extend the knee, and a palpable suprapatellar defect. In older patients, however, these signs may be obscured by swelling, pain-related guarding, reduced baseline mobility, or concomitant osteoarthritis. The key practical point is that an inability to perform active knee extension after even minor trauma should not be attributed to degenerative disease until extensor mechanism disruption has been actively excluded.

In the present case, degenerative knee disease likely contributed to the initial diagnostic error. Radiographic findings such as patella baja may support the diagnosis, although these signs are not always prominent in the acute setting [2,3]. Delayed recognition has been associated with quadriceps atrophy, extensor lag, stiffness, and slower functional recovery [7,8]. The clinical examination should therefore specifically document active extension, straight-leg raise ability, and the presence or absence of a suprapatellar gap.

Role of imaging

Imaging is most useful when clinical findings are incomplete or equivocal. Plain radiographs may reveal indirect signs of extensor mechanism disruption, but they cannot assess tendon integrity directly. MRI remains highly informative for soft-tissue evaluation, although it may delay treatment or be unnecessary when the diagnosis is clinically evident. In the present case, musculoskeletal US offered a rapid bedside-compatible alternative, demonstrating tendon discontinuity, proximal retraction, associated hematoma, and dynamic loss of continuity during attempted quadriceps contraction [3,24]. This allowed prompt bilateral confirmation and surgical planning without waiting for MRI.

Surgical management

The operative goal in complete BQTR is restoration of extensor mechanism continuity and reliable active knee extension [2,3,8]. Acute repairs are commonly performed using either transosseous patellar tunnels or suture anchors, with favorable outcomes reported for both approaches when tissue quality and timing permit. In this case, transosseous repair was selected because it allowed secure tendon reinsertion using a familiar and reproducible technique. Simultaneous bilateral surgery by two teams was chosen not to introduce a different repair principle, but to reduce total operative and anesthesia time.

By contrast, delayed or chronic ruptures present substantially greater technical challenges. Tendon retraction, scar formation, poor tissue quality, and quadriceps shortening may require more complex procedures such as V-Y lengthening, quadriceps advancement, graft augmentation, or other reconstructive techniques [4,7,25,26]. Despite a one-week delay in diagnosis, our patient remained suitable for primary repair without augmentation or lengthening procedures.

Rehabilitation considerations

Rehabilitation after QT repair must balance protection of the tendon-bone repair with prevention of stiffness, deconditioning, and loss of independence. This balance is especially difficult in bilateral injuries because both extensor mechanisms are compromised at the same time. The postoperative protocol in this case therefore combined brace protection, controlled progression of knee flexion, and early supervised weight-bearing.

Available evidence suggests that carefully supervised early mobilization can improve functional recovery without compromising tendon healing when fixation is stable [1,13]. In geriatric patients, early mobilization also helps reduce thromboembolic, pulmonary, and general functional complications related to prolonged bed rest. Social factors influenced the rehabilitation pathway in this patient: because she lived alone and lacked sufficient outpatient support, continued inpatient rehabilitation was required despite an otherwise uncomplicated early postoperative course.

Comparison with existing literature

Published data on simultaneous BQTR in elderly women remain scarce and are largely limited to isolated case reports. This limits the strength of conclusions regarding optimal surgical technique, rehabilitation strategy, and expected long-term recovery. The contribution of the present case is therefore practical rather than definitive: it illustrates how osteoarthritis can obscure the diagnosis, how US can accelerate confirmation, and how single-stage bilateral repair may restore early mobility in a carefully selected elderly patient.

Limitations

This report has several limitations. It describes a single case, which limits generalizability, and follow-up was restricted to the early postoperative period, so long-term functional outcome remains unknown. MRI was not performed; however, the diagnosis was clearly established through clinical examination and musculoskeletal US. Functional assessment was limited to early Lysholm Knee Scoring Scale values, without longer-term patient-reported outcome measures. Despite these limitations, the case provides clinically relevant insight into recognition and early management of a rare bilateral extensor mechanism injury.

## Conclusions

Simultaneous BQTR should be considered in older patients who present with sudden bilateral knee dysfunction and inability to actively extend the knees, even when low-energy trauma and pre-existing osteoarthritis suggest a more benign explanation. Careful clinical examination remains decisive, and musculoskeletal US can rapidly confirm the diagnosis when tendon integrity is uncertain. In the present case, transosseous single-stage bilateral repair followed by protected early rehabilitation restored functional ambulation despite a one-week diagnostic delay. The case supports a pragmatic management pathway: suspect the injury clinically, confirm it promptly, repair complete ruptures without unnecessary delay, and begin supervised mobilization as soon as fixation stability allows.
